# Using Optimal Land-Use Scenarios to Assess Trade-Offs between Conservation, Development, and Social Values

**DOI:** 10.1371/journal.pone.0158350

**Published:** 2016-06-30

**Authors:** Vanessa M. Adams, Robert L. Pressey, Jorge G. Álvarez-Romero

**Affiliations:** 1 Research Institute for the Environment and Livelihoods, Charles Darwin University, Darwin, NT, Australia; 2 Australian Research Council Centre of Excellence for Coral Reef Studies, James Cook University, Townsville, QLD, Australia; 3 School of Biological Sciences, University of Queensland, Brisbane, QLD, Australia; Auburn University, UNITED STATES

## Abstract

Development of land resources can contribute to increased economic productivity but can also negatively affect the extent and condition of native vegetation, jeopardize the persistence of native species, reduce water quality, and erode ecosystem services. Spatial planning must therefore balance outcomes for conservation, development, and social goals. One approach to evaluating these trade-offs is scenario planning. In this paper we demonstrate methods for incorporating stakeholder preferences into scenario planning through both defining scenario objectives and evaluating the scenarios that emerge. In this way, we aim to develop spatial plans capable of informing actual land-use decisions. We used a novel approach to scenario planning that couples optimal land-use design and social evaluation of environmental outcomes. Four land-use scenarios combined differences in total clearing levels (10% and 20%) in our study region, the Daly Catchment Australia, with the presence or absence of spatial precincts to concentrate irrigated agriculture. We used the systematic conservation planning tool Marxan with Zones to optimally plan for multiple land-uses that met objectives for both conservation and development. We assessed the performance of the scenarios in terms of the number of objectives met and the degree to which existing land-use policies were compromised (e.g., whether clearing limits in existing guidelines were exceeded or not). We also assessed the land-use scenarios using expected stakeholder satisfaction with changes in the catchment to explore how the scenarios performed against social preferences. There were a small fraction of conservation objectives with high conservation targets (100%) that could not be met due to current land uses; all other conservation and development objectives were met in all scenarios. Most scenarios adhered to the existing clearing guidelines with only marginal exceedances of limits, indicating that the scenario objectives were compatible with existing policy. We found that two key stakeholder groups, agricultural and Indigenous residents, had divergent satisfaction levels with the amount of clearing and agricultural development. Based on the range of benefits and potential adverse impacts of each scenario, we suggest that the 10% clearing scenarios are most aligned with stakeholder preferences and best balance preferences across stakeholder groups. Our approach to scenario planning is applicable generally to exploring the potential conflicts between goals for conservation and development. Our case study is particularly relevant to current discussion about increased agricultural and pastoral development in northern Australia.

## Introduction

Ecosystems provide an array of goods and services that contribute to human well-being [1, 2]. However, land-use decisions alter ecosystems, ranging from minor changes to complete transformation of natural and human-dominated landscapes [2–4]. For example, conversion of native vegetation to intensive agriculture can increase the provision of services such as food, forage, medicines, and bioenergy, and thus contribute to livelihoods, but may compromise other services, such as nutrient cycling, water quality, and carbon sequestration [5, 6]. Likewise, expansion of intensive land uses can have negative impacts on other natural values of ecosystems, including loss of biodiversity [7] and local cultural values [8].

Planning for multiple land uses thus requires navigating trade-offs between social, economic, and conservation outcomes, all of them important aspects of human well-being, arising from different possible futures [9]. Scenario planning [10] is one approach that is becoming more widely used to assess the impacts of different mixes of future land uses. The benefits of scenario planning include: 1) incorporating uncertainty by exploring outcomes associated with multiple plausible futures, thus assisting in the development of more robust policies [10, 11], and 2) supporting participatory processes where scenarios are analyzed and discussed, thereby building a shared understanding across diverse stakeholder groups [12, 13].

There is a growing number of spatially explicit models of land-use change for scenario planning [5, 14, 15–17]. While these studies assess trade-offs between economic and ecological values (e.g., land production, carbon storage, species diversity), they do not incorporate preferences of local stakeholders related to their well-being and fall short of providing local-scale policy recommendations. Stakeholder preferences are a critical aspect of successful planning, providing legitimacy and ownership of planning outputs and improving uptake and implementation [18]; however stakeholder preferences have been considered infrequently when assessing potential trade-offs in land uses (but see [19, 20, 21]). Measuring indicators related to well-being in scenario planning can make outputs more relevant to stakeholders and contribute to the process of incorporating stakeholder preferences to evaluate potential trade-offs and build consensus around a single land-use option [22].

Given the acknowledged value of understanding and including preferences into the planning process, there is a growing body of literature that demonstrates ways of eliciting and incorporating this information. Scenario planning studies have typically incorporated stakeholder preferences in terms of stakeholder-defined drivers (e.g., market forces or regulations) of land-use change [5], or in terms of economic performance of scenarios [12]. Approaches to including stakeholder values and preferences in spatial planning include mapping of preferences [21, 23], participatory planning methods to capture stakeholder preferences interactively [19, 20, 24], and post-planning analysis of impacts based on changes in indicators [13]. While some of the above-mentioned studies include stakeholder preferences in terms of mapped values or impacts on economic commodities, there remains a gap in approaches for incorporating local preferences–identified here in terms of well-being factors and satisfaction with specific socioeconomic and environmental changes–to both develop and evaluate regional scenarios.

We consider the incorporation of local preferences, of people directly affected by land-use changes, as necessary to develop spatial plans capable of informing actual land-use decisions. Thus, the primary aim of our study is to present one approach for incorporating local preferences into both the objectives for scenarios as well as the evaluation criteria for assessing the scenarios’ performance. Our study therefore contributes to two of the approaches to including stakeholder values and preferences in spatial planning, namely eliciting preferences to inform plan objectives and analysis of scenarios based on indicators defined and parameterized by stakeholder surveys. We demonstrate this joint approach to design and evaluation of multiple land-use scenarios as a key component of developing a conservation and development plan for the Daly River catchment, in Australia’s Northern Territory. Advances in scenario planning are likely to be crucial for resolving debate around the Australian Government’s recent emphasis on expanding agricultural and pastoral development in northern Australia [25]. More broadly, our study demonstrates how spatial planning can be combined with scenario analysis and perceptions of well-being of affected stakeholders to shape choices between alternative futures for regions.

## Methods

### Study region

**Fig 1:**
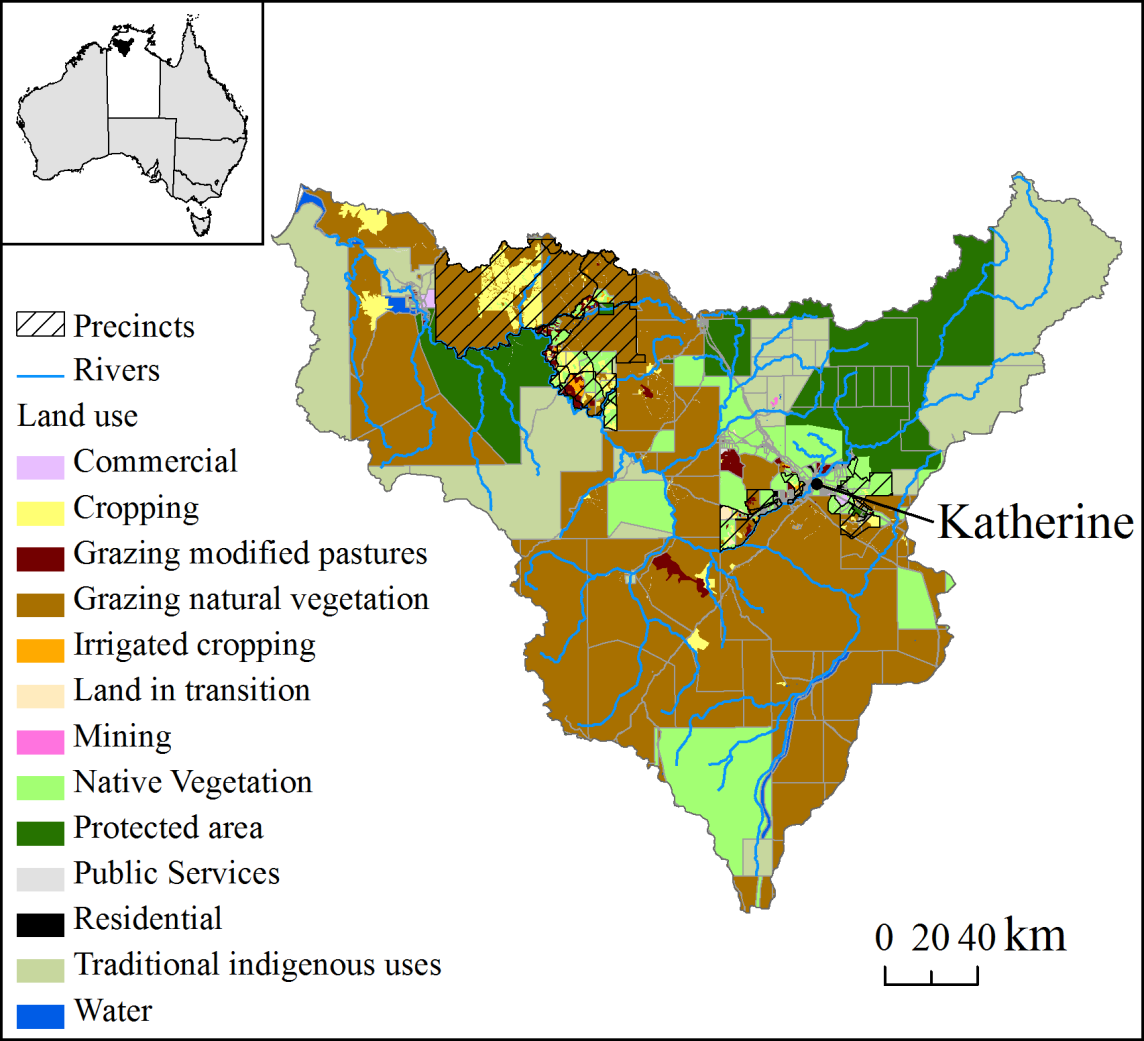
Daly catchment property boundaries and land use as defined by ABARES Land use mapping. Inset shows the Northern Territory (white) and the Daly catchment (black).

The study region is the whole of the Daly River catchment in the Northern Territory (NT), Australia (Fig 1), which is approximately 5.2 million ha, extending from the coastline southwest of Darwin to 250 km inland. Approximately 13% of the catchment is currently protected by national parks and Indigenous protected areas, while about 6% has been cleared of native vegetation (or at least the native overstorey) for different intensive land uses such as cropping and modified pastures (Fig 1). The Daly catchment has been recognized both for its high conservation values and potential for future agricultural development due to a unique combination of suitable soils, year-round water supplied by large aquifers and the perennial Daly River, and suitable climatic conditions (adequate rainfall during the growing period) for rainfed crops [26]. Also, an emerging market for offsets to reduce carbon emissions based on improved fire management of tropical savannas could provide an important commercial opportunity for grazing properties and Indigenous lands in the region [27, 28].

Conservation values in the catchment include five sites of conservation significance identified by the NT Government [29], extensive rainforest galleries, and habitats for important wildlife populations, especially of fish, turtles, and waterbirds [30]. The catchment is recognized nationally and internationally for its high ecological values. The estuary and lower floodplains meet waterbird-based criteria for listing as a Ramsar Wetland of International Importance [31]. The middle and upper parts of the catchment contain national parks and Indigenous protected areas with widely recognized importance for biodiversity, cultural values, and scenery. The land and water systems of the Daly also sustain important cultural, spiritual, and socioeconomic activities for Indigenous and non- Indigenous people [9, 32].

### Planning process

**Fig 2:**
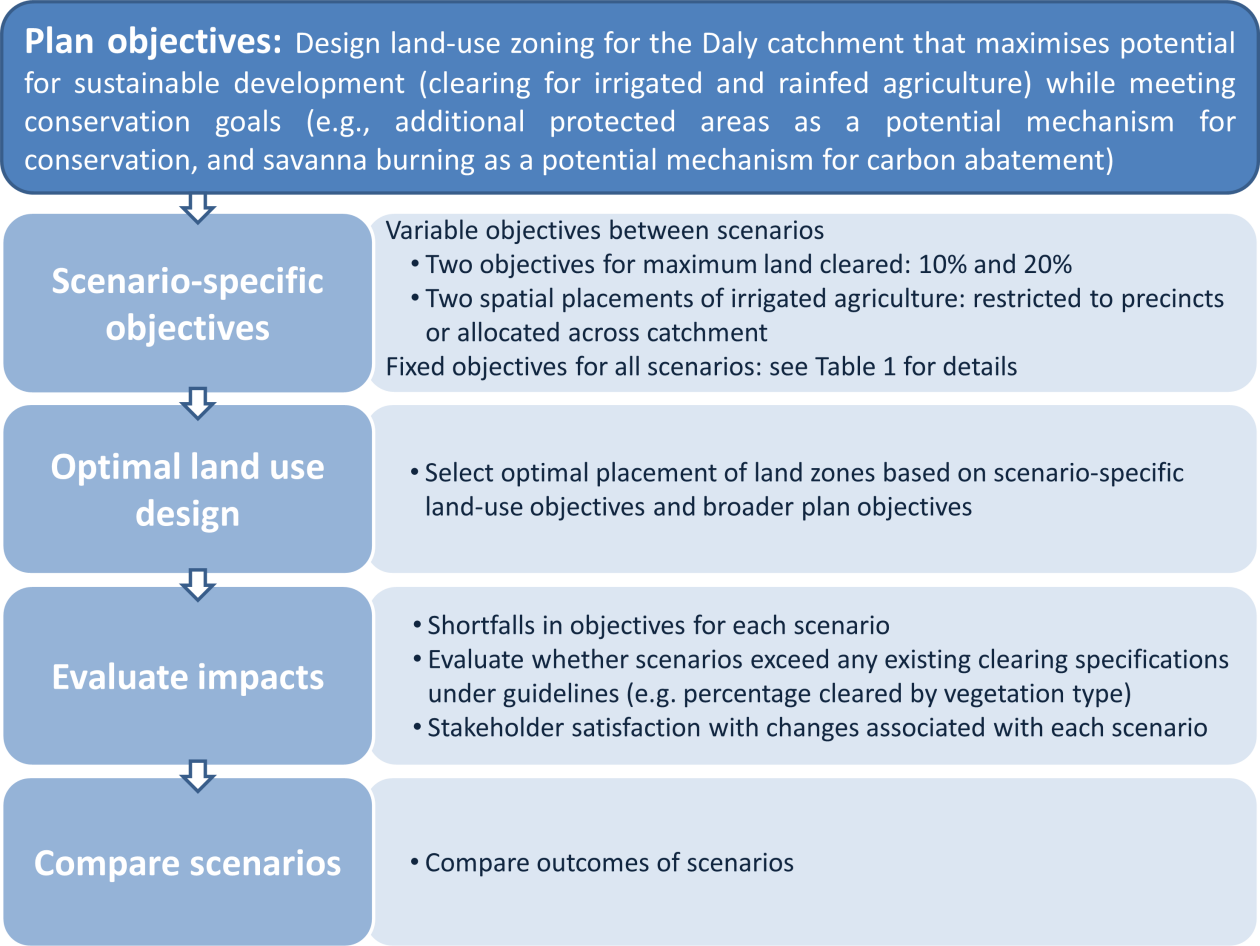
Process for the design and evaluation of land-use scenarios to support the development and conservation plan for the Daly River catchment.

In response to potentially competing interests in the management of the Daly catchment (e.g., conservation, agriculture, recreation), the former Daly River Management Advisory Committee (DRMAC) commissioned a development and conservation plan for the catchment in 2011. The planning process was designed by us in collaboration with DRMAC with the intent of producing an agreed-upon land-use scenario to support the ongoing adaptive planning and management of decisions about natural resources over management relevant time-frames of 5–10 years [22]. The three-year planning process (Fig 2) broadly follows the systematic conservation planning framework outlined by Pressey and Bottrill [33]. It was agreed that a scenario-planning approach would be used in which a number of possible land-use futures would be designed, evaluated, and delivered to DRMAC (Fig 2). Selection and staged implementation of the final land-use scenario as the preferred development and conservation plan was to be managed adaptively [34] by appropriate decision makers such as the NT Government or catchment advisory committees.

The land-use scenario to emerge from this process was thus seen as an initial guide for land-use decisions in the short-term (5–10 years), acknowledging the potential for ongoing participation and adaptation of the scenario with changes to background information, the expectations of local stakeholders, and political constraints and opportunities. The proposed planning process was therefore an initial roadmap to involving stakeholders in guiding ongoing and future planning in the region. Consequently, the proposed planning strategy assumes that implementation would involve refinement of objectives, progressive updating of the plan, and evaluation of ongoing resource-use decisions, over the plan’s lifetime of 5–10 years. In the following sections, we provide details for each of the planning stages identified in Fig 2.

Both the design and evaluation of scenarios were strongly influenced by stakeholders in the Daly catchment. The four potential future configurations of land uses that defined our scenarios were shaped by objectives related to land uses, originally identified by DRMAC and checked against stakeholder preferences. Our evaluation of the scenarios depended on DRMAC’s objectives related to biodiversity and on surveys of catchment residents to elicit changes in satisfaction corresponding to changes reflected in the scenarios.

### Plan objectives

The plan objectives were set in collaboration with DRMAC. Defining objectives involved first describing a number of broad, qualitative goals and then translating these into quantitative objectives to inform land-use scenarios (for a full discussion of the objective setting process see [22]). The plan objectives cover development (e.g., maximum percentage of catchment cleared for agriculture), further protection of conservation features (e.g., protection of 17% of all vegetation types), and supporting alternative commercial opportunities (e.g., carbon offsets) (Table 1). A stakeholder engagement process was designed in collaboration with DRMAC to inform the plan objectives and ensure the plan outcomes were aligned with stakeholder values. The engagement process extended over the duration of the planning process (2012–2014) and included community forums, focus groups, and a survey to all catchment residents (for full details of process and results of engagement components see [22]). The stakeholder engagement ensured that all plan objectives identified by DRMAC were also aligned with stakeholder values. Therefore, the plan objectives remained broadly the same as those initially identified by DRMAC, with the exception of two objectives (maximum clearing and agricultural precincts) that were varied across scenarios.

**Table 1:** Qualitative goals and associated quantitative objectives for the land-use scenarios. Qualitative goals are in bold. Under each goal are listed the associated actions, available mapped features for interpretation of goals, quantitative objectives related to mapped features, and the applicable land-use zone. N/A indicates that there were no available map products.

	Qualitative goals and related actions	Spatially-defined features	Quantitative objectives	Applicable zone
**Maintain water flow of rivers**				
	Manage water extraction	N/A	None defined. Instead, performance of land-use scenarios was assessed quantitatively in relation to water-extraction levels with a water-management tool, reflecting principles of an existing water plan and associated extraction limits [46]	N/A
**Maintain fish populations**				
	Manage water extraction	N/A	None defined. Instead, performance of land-use scenarios was assessed quantitatively in relation to water-extraction levels with a water-management tool, reflecting principles of an existing water plan and associated extraction limits [46]	N/A
	Protect important stream reaches	Predicted occurrences of 106 fish species [30]	17% of occurrences of each species (reflecting CBD targets, [41])	Protected
**Protect biodiversity**				
	Protect Sites of Conservation Significance	Sites of Conservation Significance^1^ (5) [29]	100% of sites (reflecting expert opinion on value of sites)	Protected
	Protect representative portions of species’ habitats	Vegetation mapping (104 types) and bioregion boundaries (9) [47]	17% of extent of each vegetation type; 17% of each bioregion (reflecting CBD targets, as above)	Protected
	Protect representative portions of species’ occurrences	Predicted occurrences of fish (106), bird (106), and turtle (13) species [30]	17% of occurrences (reflecting CBD targets, as above)	Protected
	Protect wetlands	Mapped wetlands (4) [47]	100% of wetlands (reflecting expert opinion on value of sites)	Protected
	Protect rainforest galleries	Mapped rainforest (1) [47]	100% of rainforests (reflecting expert opinion on value of sites)	Protected
	Manage fire threats to biodiversity	Expected savanna burning abatement^2^ [48]	10% of abatement (measured in metric tonnes of carbon dioxide equivalents, [48,49]); constrained to Indigenous land which is the primary tenure engaged in abatement activities and the associated offset market.	Savanna burning
**Increased development and diversification of land uses and industries**				
	Clear suitable land for agricultural use	Land suitability categories [26]	100% of highly suitable land cleared with a maximum of 20% clearing across the whole catchment (reflecting clearing guideline catchment limit, [36])	Annual Irrigation, Perennial Irrigation, Rainfed Cropping^4^
	Encourage diversified land uses	Land suitability categories [26]	Land cleared for new agricultural developments to be distributed across different types of crops: 40% perennial irrigation of new clearing, 20% annual irrigation of new clearing, 40% rainfed cropping of new clearing. For the maximum 10% cleared scenarios this corresponds to total catchment cleared of: 2% perennial irrigation, 1% annual irrigation and 2% rainfed cropping. For the maximum 20% cleared scenarios this corresponds to total catchment cleared of: 6% perennial irrigation, 3% annual irrigation and 6% rainfed cropping.	Annual Irrigation, Perennial Irrigation, Rainfed Cropping
	Build critical mass of human populations and businesses to support agricultural development	Agricultural precinct boundaries^3^	Promote spatially-concentrated agricultural development in precincts to support critical mass for communities and infrastructure.	Annual Irrigation, Perennial Irrigation, Rainfed Cropping
	Support alternative commercial activities such as carbon offsets	Expected savanna burning abatement^2^ [48]	10% of abatement (measured in metric tonnes of carbon dioxide equivalents, [48]); constrained to Indigenous land which is the primary tenure engaged in abatement activities and the associated offset market.	Savanna burning
**Proper use of land and natural resources**				
	Constrain land clearing to suitable land	Land suitability categories [26]	Allow clearing only on suitable land and allocate lands to best agricultural land uses (annual irrigation, perennial irrigation or rainfed crops)	Annual Irrigation, Perennial Irrigation, Rainfed Cropping

^1^The Northern Territory Government undertook an assessment of conservation and heritage values and identified 67 of the most important sites for biodiversity conservation across the Territory, some of which are in the Daly catchment. By definition, these sites need adequate protective management.

^2^Savanna burning is an approved methodology for greenhouse-gas abatement under the Carbon Farming Initiative (CFI) in Australia. It involves fire management to reduce the extent of fires and adjust their timing by burning earlier in the dry season, thereby reducing the total emissions associated with annual fires. Current enrolled properties for savanna-burning credits under the CFI are all Indigenous.

^3^Precinct boundaries were developed in consultation with experts and reflect existing land zonings and agricultural land use in the catchment.

^4^New clearing allocated to three types of cleared land: annual irrigation, perennial irrigation and rainfed cropping. We chose to allocate new clearing explicitly to these three zones as land suitability varies across uses as well as water requirements and productivity. As such it is useful for decision makers to know which type of land use is allocated to a planning unit.

### Scenario-specific objectives

Four land-use scenarios were developed by varying two objectives related to development (Table 2): total land cleared across the catchment; and spatial constraint on location of new irrigated agriculture (constrained to precincts or unconstrained). The objectives for conservation (i.e. protection of native vegetation, native species, and sites of conservation significance) and ecosystem services (i.e. abatement of carbon emissions through improved savanna burning) were constant across scenarios.

**Table 2:** Final set of land-use scenarios.

		Location of irrigated agriculture	
		Unconstrained	Precincts only
**Objectives for maximum clearing of native vegetation**	**10%**	(a) Scenario 1: Maximum of 10% clearing across the catchment; no constraints on location of irrigated agriculture	(b) Scenario 2: Maximum of 10% clearing across the catchment; new clearing for irrigated agriculture constrained within precincts
	**20%**	(c) Scenario 3: Maximum of 20% clearing across the catchment; no constraints on location of irrigated agriculture	(d) Scenario 4: Maximum of 20% clearing across the catchment; new clearing for irrigated agriculture constrained within precincts

DRMAC set a ceiling of 20% of total clearing across the catchment to reflect the existing clearing guidelines (Table 2) [35, 36]. However, a process of stakeholder engagement undertaken to support the development of the plan revealed that many respondents preferred maximum clearing levels of 10% but would accept 20% with steadily decreasing satisfaction beyond 20% [22]. We therefore chose to explore two objectives for maximum land clearing: 10% and 20% (Table 2).

Likewise, DRMAC set a goal to build critical mass for communities and infrastructure to support pastoral and agricultural development, identifying an associated action of constraining irrigated agriculture to existing agricultural precincts in the catchment, in line with national policies of intensifying agricultural development within precincts [37]. The Douglas Daly and Katherine regions of the catchment are informally recognized as existing precincts based on their soil suitability and water resources [38]. However, in order to prioritize the expansion of agriculture within these zones we mapped precinct boundaries for the two regions in collaboration with relevant government and industry partners. We explored two precinct scenarios: 1. irrigated agriculture constrained within precinct boundaries, with only rainfed cropping outside of these boundaries; and 2. no constraints on the location of irrigated agriculture (Table 2).

### Optimal land-use design

We used a decision-support tool, Marxan with Zones [39], to generate alternative configurations of land uses that achieved our objectives for conservation and development. Marxan with Zones is a multiple-use planning version of Marxan used to identify configurations of land or water uses that achieve specified plan objectives while minimizing cost.

For our land-use scenarios we used micro-catchments as our planning units (19,402 units, mean area 270 ha) for prioritization. Using micro-catchments was necessary to target fish occurrences related to one of our goals for biodiversity protection. We identified five land uses (hereafter zones), three of which contributed to development and associated clearing objectives (perennial irrigation, annual irrigation, and rainfed cropping), one zone associated with alternative livelihoods through carbon offsets (savanna burning) which contributed to both development and conservation goals, and one zone for conservation goals (protection) (see Table 1). Due to limitations in current land-use mapping we did not have a consistent map of how cleared land is currently used across irrigation and cropping zones. Our scenarios therefore considered only changes in land-use of currently uncleared land. Planning units not associated with any of our five zones (termed ‘available’) could be allocated by Marxan with Zones to one of the five zones. We identified which zones could potentially be allocated to each planning unit based on existing land use, tenure, agricultural suitability, and precinct boundaries. The ‘savanna burning’ zone was constrained to Indigenous land because this is the primary tenure engaged in abatement activities and the associated offset market [27, 28]. Planning units not allocated by Marxan with Zones to one of our five zones were considered to remain “available”, effectively indicating no change in land use.

We included plan objectives for each zone relating to 348 spatially-defined conservation features for protection, land-use suitability, and potential for carbon abatement through modified savanna burning (see Table 1 for data sets, objectives, and applicable zones for achieving objectives).

We set the costs of planning units equal to their areas for three reasons. First, much of the land-use plan will be implemented through land-use policy changes and not acquisition of land. For example, zoning an area for irrigated cropping would require changes to leasehold land regulations to allow current owners to engage in new commercial activities rather than the purchase of land by existing or new landholders. Second, there are multiple pathways, with different cost implications, by which some planning units could be converted to a new land use. For example, areas could be protected by covenants or management agreements or acquisition by government or private conservation organizations [40]. Third, regional planning with costs inevitably involves proxies or models of selected cost components that are unlikely to reflect the actual costs of land-use transitions on the ground, necessitating fine-tuning of plans as implementation proceeds and actual costs become apparent [34].

Setting cost equal to area allowed us to constrain the total area selected for zones without attempting to otherwise specify the costs of zones. This ensured that the plan was efficient in terms of total area allocated to each land-use zone, and recognized that implementation would involve iterative modification of the initial plan as on-ground investigations of individual areas yield more accurate data on biodiversity, land-use suitability, costs, and other key variables. While our approach ensures that land uses are placed on the most suitable land, it does not incorporate economic returns from alternate land uses. Our approach could be extended to include economic returns on land use to maximize conservation objectives for given levels of economic returns if appropriate data were available (e.g., [15]). We parameterized Marxan with Zones to allow aggregation of planning units based on shared boundaries between planning units to achieve a realistic (non-fragmented) representation of land uses. To control the aggregation of selected units, we identified the zone boundary cost (similar to the Boundary Length Modifier in Marxan) such that spatial aggregation was balanced with the cost of aggregation [39]. For each scenario we used the Marxan with Zones ‘best’ solution (i.e. a near-optimal configuration of zones that achieved objectives with the least amount of land-use change). We compared the maps of spatial solutions across scenarios and the total area allocated to each land-use per scenario. In order to investigate the variability of spatial allocation of land uses to particular subcatchments we also calculated the frequency of selection for each subcatchment across the four scenarios for irrigated and rainfed cropping (see S1 and S2 Figs for details).

### Evaluate impacts and compare scenarios

For each scenario, we compared the number of objectives met based on summary statistics for the best solution. There are existing clearing guidelines for the Daly catchment that set a catchment-wide clearing limit of 20%, with additional nested limits for vegetation types (30%) and sub-catchments (40%). Our scenarios were designed to meet upper limits of total catchment clearing of either 10% or 20% (Table 2). However, instead of limiting clearing by vegetation type and sub-catchment, we ensured that each vegetation type and sub-catchment had a minimum percentage protected (17%, from the Aichi targets, see [41]). Therefore, for each land-use scenario, we calculated the number of vegetation types and sub-catchments in which clearing exceeded the respective limits of 30% and 40% specified in the clearing guidelines (for further detail, see [35]). Where a clearing limit had been exceeded for a vegetation type or sub-catchment, we classified exceeded limits as marginal (greater than limit but less than 50% cleared) or major (greater than 50% cleared).

We also wanted to understand how changes in the catchment associated with the land-use scenarios would affect stakeholders over a range of relevant social, commercial, and environmental factors. To do this we used the data from surveys of satisfaction reported by [22] (surveys were sent to all catchment residents, with a representative sample of 209 responses received). The previous study elicited changes in satisfaction in response to a variety of potential changes in environmental characteristics of the Daly catchment. Changes in satisfaction were summarised for all respondents and for two sub-groups: Indigenous respondents, and respondents who earn an income from agricultural land uses (in this case pastoralism and cropping) [22].

**Fig 3:**
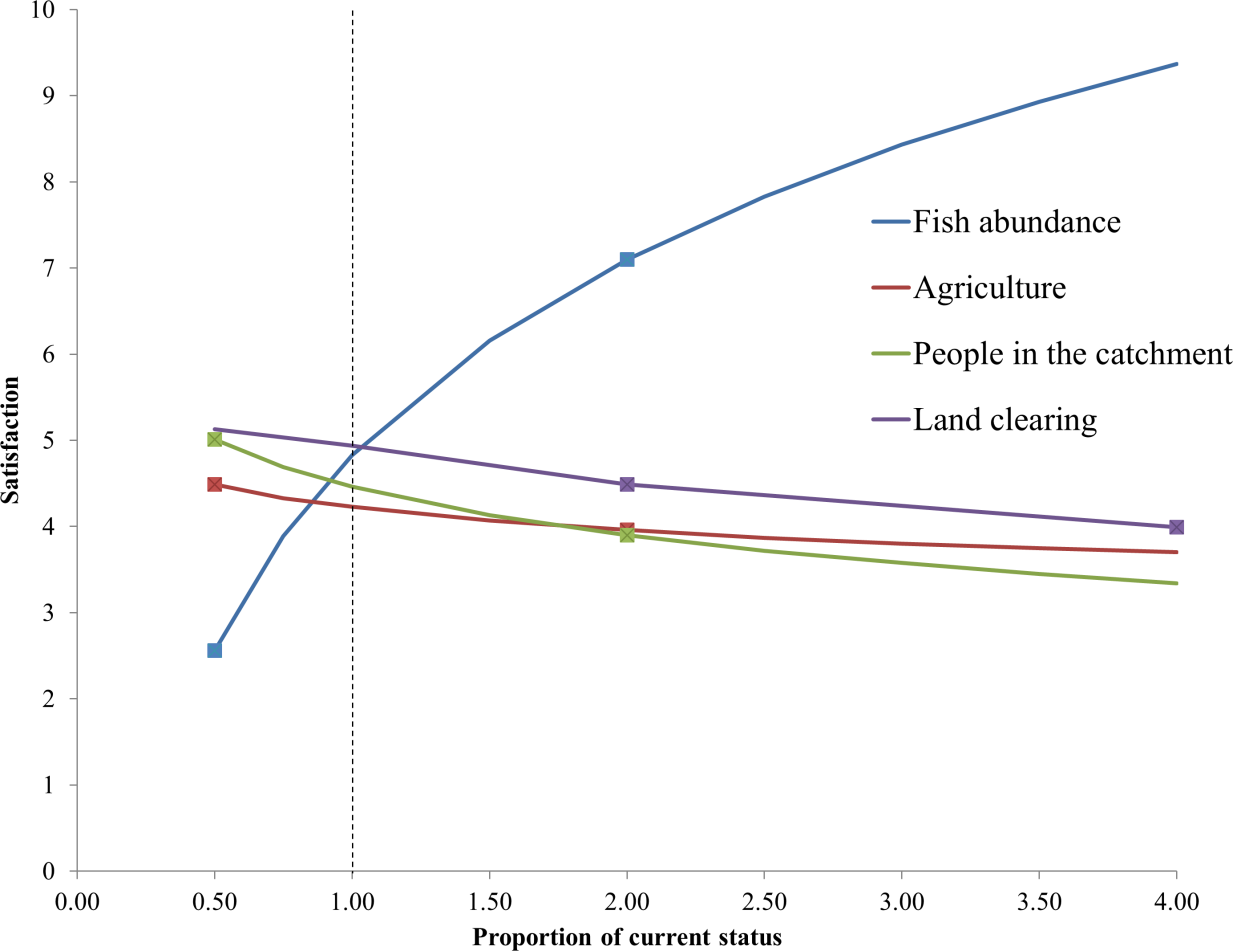
Changes in satisfaction of residents in relation to socioeconomic and environmental factors. Values shown here are based on means across all respondents from the survey of Adams et al. [22]. Square markers indicate reported satisfaction levels elicited directly from the survey. Changes in satisfaction relative to changes in the catchment outside the elicited ranges (reported satisfaction levels shown with square markers) are based on linear extrapolation. The dashed line indicates the current state of factors.

For the present study, we began with the stated averages for satisfaction with catchment changes in the Daly reported by [22]. We related the changes in land clearing for our scenarios to catchment changes including water levels of the Daly River in the dry season, amount of infrastructure, amount of agriculture, number of people living in the catchment, number of fish in the river, and amount of land cleared (Table 3). Where possible we used the exact stated average satisfaction for these factors from the survey results. However, our scenarios resulted in predicted changes in some factors that were outside the range of changes presented to residents of the Daly catchment in the previous study. For these factors, we used linear extrapolation to estimate satisfaction ratings for other states of the catchment (e.g., four times as much agriculture). A linear function was chosen after checking that the reported satisfaction levels in positive and negative changes in factors were similar in magnitude (i.e. no non-linear responses to positive and negative changes were detected). Similar analyses were done separately for mean changes reported by Indigenous and agricultural respondents. Extrapolated curves from stated satisfaction from all respondents are in Fig 3.

**Table 3:** Description of assumed relationship between percentage clearing scenarios and social, commercial and environmental factors. (a) 10% clearing scenarios factors. (b) 20% clearing scenario factors.

**(a) 10% Clearing**	**Description of relationship**
Water level dropped in the Daly (dry season)	An increase in agriculture will be accompanied by increased water extractions and decreased water flows in the dry season [9].
Twice the infrastructure	During our consultation with DRMAC the relevant government departments indicated that increased agriculture would trigger investment (by both government and industry) to double existing infrastructure such as paved roads, electricity infrastructure (e.g. most farms still run on diesel generators), and mobile phone towers (e.g. most farms have only landline phone access).
Twice as much agriculture	In our 10% clearing scenario all cleared land was assigned to agricultural land uses, thus resulting in a doubling of agriculture (from ~5% to 10%).
One and a half times as many people in the catchment	Based upon the cadastral data we estimate that ~25% of properties in the catchment have agricultural land uses. We assumed a linear relationship between increased agricultural production and the labour force required. This resulted in approximately one and a half times more people living in the catchment.
Three quarters as many fish	Stoeckl et al [9] estimated ~50% decline in fish catch in simulations with approximately four times as much agriculture. We used this figure to estimate that for twice as much agriculture there would be a 25% decline.
Twice as much clearing	The current cleared land is ~5% of the catchment. Thus 10% clearing results in twice as much cleared land.
**(b) 20% Clearing**	**Description of relationship**
Water level dropped in the Daly (dry season)	An increase in agriculture will be accompanied by increased water extractions and decreased water flows in the dry season [9].
Twice the infrastructure	During our consultation with DRMAC the relevant government departments indicated that increased agriculture would trigger investment (by both government and industry) to double existing infrastructure such as paved roads, electricity infrastructure (e.g. most farms still run on diesel generators), and mobile phone towers (e.g. most farms have only landline phone access).
Four times as much agriculture	In our 20% clearing scenario all cleared land was assigned to agricultural land uses thus resulting in a quadrupling of agriculture (from ~5% to 20%).
Twice as many people in the catchment	Based upon the cadastral data we estimated that ~25% of properties in the catchment have agricultural land uses. We assumed a linear relationship between increased agricultural production and the labour force required. This resulted in approximately two times more people living in the catchment.
Half as many fish	Stoeckl et al [9] estimated ~50% decline in fish catch in simulations with approximately four times as much agriculture.
Four times as much clearing	The current cleared land is ~5% of the catchment. Thus 20% clearing resulted in four times as much cleared land.

Because Adams et al. [22] identified differences in the importance of well-being factors and satisfaction with land-use changes across stakeholder groups (particularly between Indigenous respondents and those who earned an income from agriculture), we summarized satisfaction changes for all stakeholders combined, as well as for Indigenous and agricultural stakeholders separately. The summaries indicated the direction and intensity of changes in satisfaction with each of the changes in use and condition. Our results show a positive change in satisfaction of <1 (on a 0–10 Likert scale, denoted with +), a positive change in satisfaction >1 (++), a negative change in satisfaction of <1 (-), and a negative change in satisfaction >1 (—) (for exact numerical changes in satisfaction see S1 and S2 Tables). We chose a change of 1 as the breakpoint because the majority of changes were <1 (see S1 and S2 Tables) and the Likert scale used in the survey asked respondents to report in whole integer changes.

## Results

**Fig 4:**
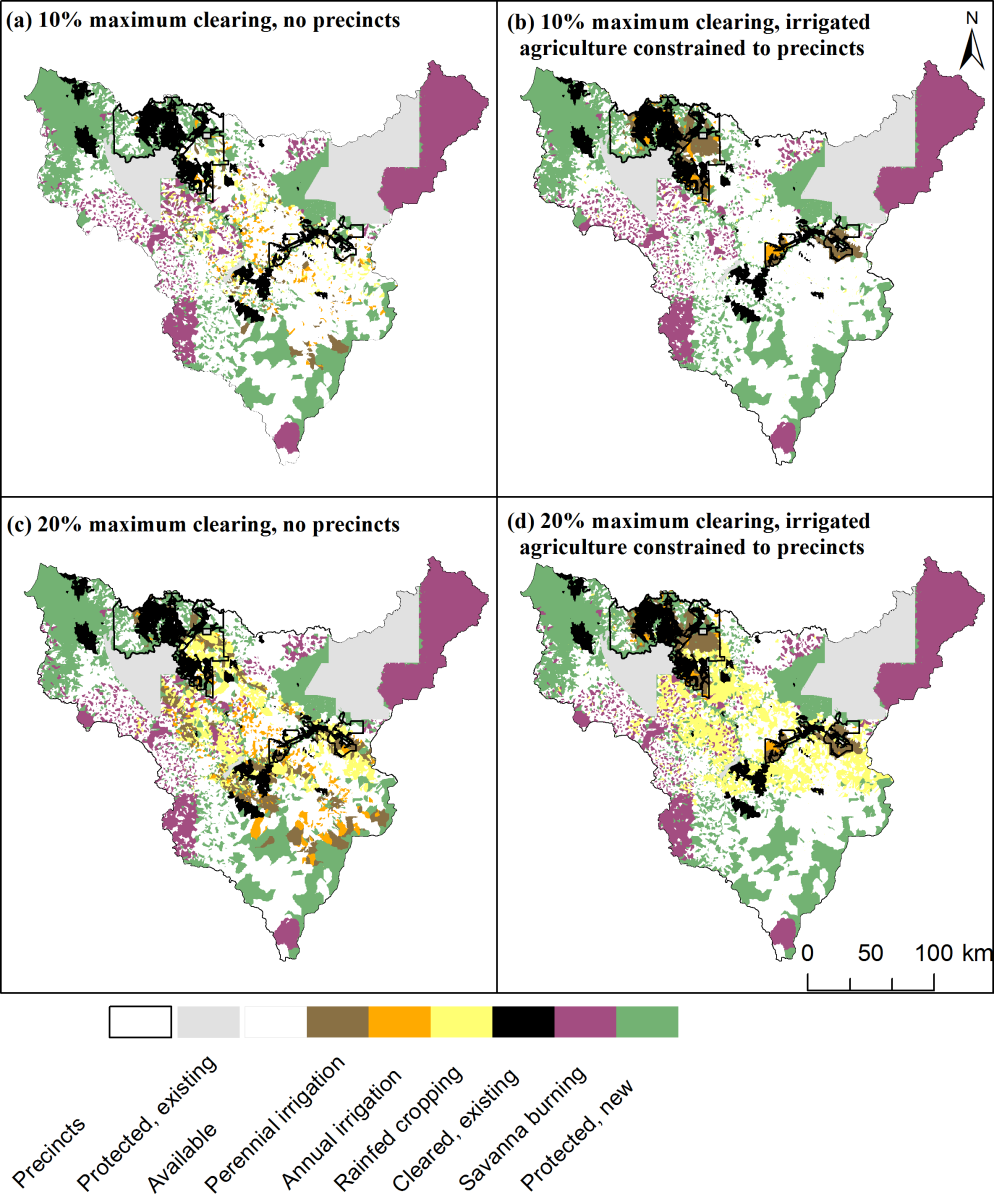
Land-use scenarios developed with Marxan with Zones. (a) scenario 1; (b) scenario 2; (c) scenario 3; (d) scenario 4.

**Fig 5:**
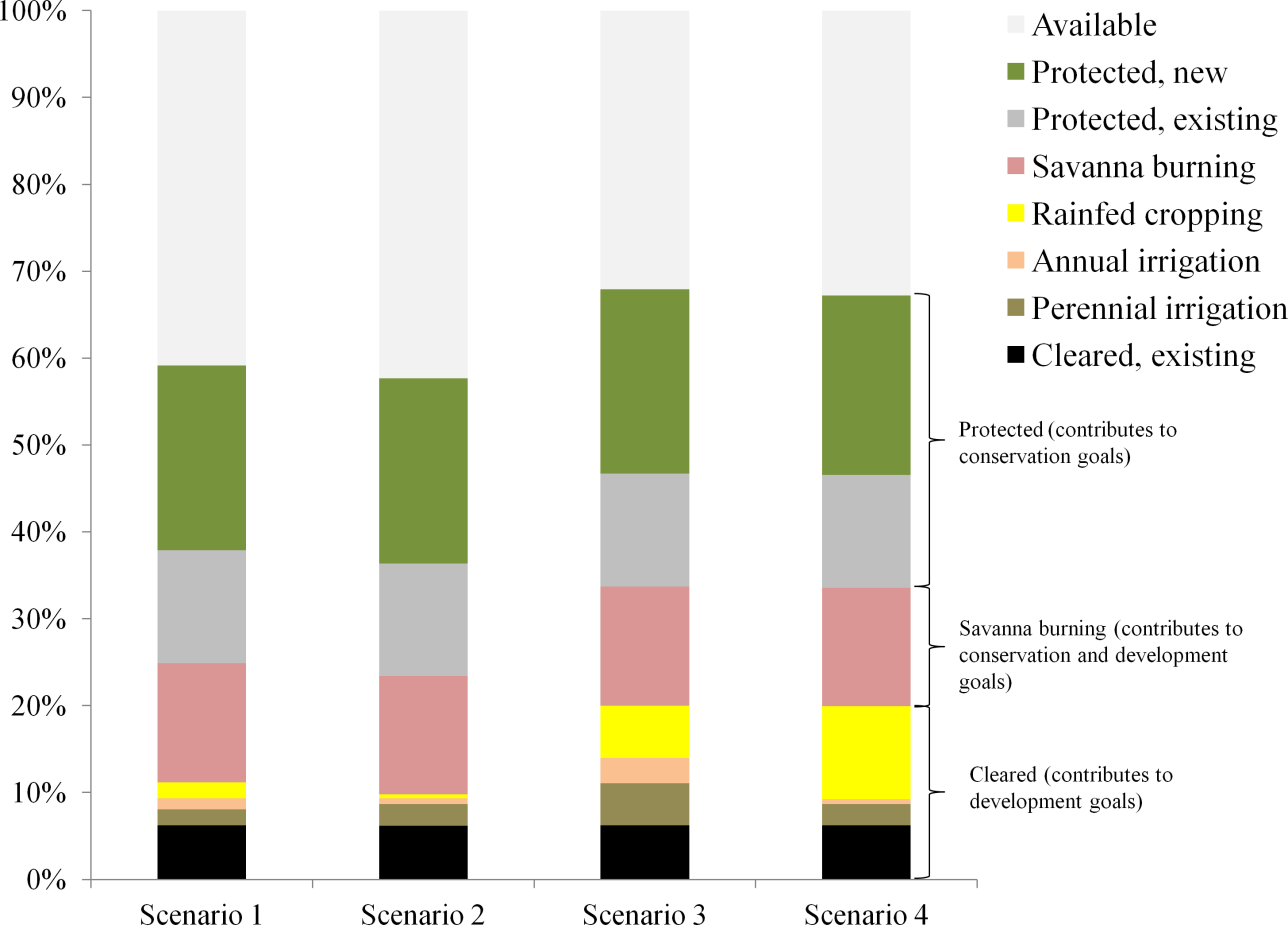
Percentage of total catchment area allocated to each land use by scenario. Colors correspond to land uses in Fig 3 and similar land uses are stacked together (protected, savanna burning, cleared).

The four land-use scenarios were spatially distinctive (Fig 4). The objectives for conservation and savanna burning were constant, resulting in very similar spatial placement (Fig 4) and quantity (Fig 5) of land selected for these uses across scenarios. About 1.10 (varying from 1.08 to 1.11) million ha were selected for protection and 0.713 (varying from 0.712 to 0.716) million ha for savanna burning. Five conservation objectives could not be met due to current land uses. These were the 100% objectives for wetlands and rainforest and for three of the five Sites of Conservation Significance: Daly River Middle Reaches, Western Arnhem Plateau, and Anson Bay. There were no other shortfalls for conservation objectives across the four scenarios.

The scenarios varied according to objectives for total clearing and location constraints for irrigated agriculture, resulting in differences in configuration and extent of agricultural land uses (Figs 4 and 5). However, there were spatial locations that were consistently selected for particular agricultural land uses across scenarios (Fig 4 and S1 and S2 Figs). For example, in the precinct scenarios, most of the Douglas Daly and eastern portion of the Katherine Precinct are allocated to perennial irrigation; in the 20% clearing scenarios, the middle portion of the catchment is allocated to rainfed cropping (Fig 4 and S1 and S2 Figs).

When clearing was unconstrained spatially (i.e. irrigated agriculture was allowed to be located outside precincts), targeted balances of land uses in new clearing (Table 1) were achieved by allocating each zone to suitable land across the catchment (Fig 4A and 4C). However, when clearing for irrigated agriculture was constrained to precincts, there were large differences in the total amount of land allocated to rainfed cropping across scenarios. Namely, the area allocated to rainfed cropping was notably larger in Scenario 1 (2%) compared to Scenario 2 (0.5%), and smaller in Scenario 3 (6%) compared Scenario 4 (10.5%) (Fig 5). The reason for these differences is that the two precincts accounted for approximately 9.5% of the catchment area which was then allocated primarily to irrigated agriculture in scenarios 2 and 4 (Fig 4). Therefore, in Scenario 2, for which maximum clearing was 10% and clearing for irrigated agriculture was constrained to precincts, only the remaining 0.5% of land available for clearing outside of precincts could be allocated to rainfed cropping, compared to the targeted 2% (Fig 5). Conversely, in Scenario 4, for which maximum clearing was 20% and irrigated cropping was constrained to the precincts, the percentage of land allocated to rainfed cropping was larger than the objective for this use (~10.5% of land available for new clearing outside of precincts was allocated to rainfed cropping compared to targeted 6%, Fig 5). Thus, while precinct constraints ensured that all irrigated agriculture was concentrated spatially to encourage economies of scale in production and infrastructure, they had consequences for the relative availability of land for other competing production uses such as rainfed cropping.

When evaluating current land uses against the clearing limits previously defined for vegetation types and sub-catchments, one sub-catchment (Green Ant Creek) had already exceeded its clearing limit, but no vegetation types had exceeded their limits [35]. Because the maximum total land clearing objectives were set to 10% and 20% in our scenarios, all scenarios fell within the overall clearing limit (i.e. 20% for the whole catchment) set by the clearing guidelines. Scenario 1 did not exceed any vegetation type or sub-catchment limits; new clearing for development in scenarios 2–4 resulted in a small number of vegetation types (up to 6 of 99) and additional sub-catchments (up to 3 of 16) exceeding the clearing limits (Table 4). Unsurprisingly, the 20% scenarios (scenarios 3 and 4) exceeded more limits than the 10% scenarios (scenarios 1 and 2). The precinct scenarios (scenarios 2 and 4) increased the number of limits exceeded compared to their counterparts without location constraints by focusing more clearing on specific vegetation types (Table 4). Exceedances to the clearing guidelines were primarily marginal (<50%); however, Scenario 4 resulted in the only major exceedance for one vegetation type and also had two major exceedances of sub-catchment limits (Table 4).

**Table 4:** Clearing limits specified by the Daly catchment clearing guidelines that were exceeded by new clearing in the Daly with each land-use scenario. Clearing limits exceeded are classified as marginal (greater than limit but less than 50%) and major (greater than 50%). The exceeded limit for Green Ant Creek sub-catchment is excluded because it was already exceeded by the current land-use configuration [35].

	Vegetation types, marginal^1^	Vegetation types, major	Subcatchments, marginal^2^	Subcatchments, major
**Scenario 1**	0	0	0	0
**Scenario 2**	1	0	0	1
**Scenario 3**	5	0	3	1
**Scenario 4**	6	1	2	2

^1^The clearing limit for vegetation types in the Daly clearing guidelines is 30%

^2^The clearing limit for sub-catchments in the Daly clearing guidelines is 40%

Changes associated with the land-use scenarios included social (population size and infrastructure), commercial (land cleared and used for agriculture), and environmental factors (dry season water level in the Daly and quantity of fish). These changes in development and associated environmental impacts resulted in mainly negative changes in satisfaction, with the exception of increased infrastructure (Table 5). For example, a land-use scenario with 10% of the land cleared for development results in a doubling of cleared land and agriculture with accompanied pressure on water resources (resulting in a drop in water levels in the Daly and fish numbers) and increases in population and infrastructure to support the development. For changes in agricultural land use and clearing amounts there was a divergence in satisfaction for Indigenous and agricultural stakeholders: Indigenous stakeholders reported negative changes in satisfaction while agricultural stakeholders reported positive changes in satisfaction.

**Table 5:** Changes in satisfaction level of stakeholders associated with changes in social, commercial and environmental factors. Changes are relative to the current status of factors in the catchment as they relate to (a) 10% maximum clearing scenarios (scenarios 1 and 2) and (b) 20% maximum clearing scenarios (scenarios 3 and 4). + indicates a positive change in satisfaction of <1 (on a 0–10 likert scale), ++ indicates a positive change in satisfaction >1,–indicates a negative change in satisfaction of <1,—indicates a negative change in satisfaction >1 (see S1 and S2 Tables for actual Likert values). Grey cells indicate that that change was not relevant to the scenario (e.g., four times as much agriculture does not apply to 10% maximum clearing but does apply to the 20% maximum clearing scenario).

	(a) 10% maximum clearing scenarios			(b) 20% maximum clearing scenarios		
Catchment changes associated with land-use scenarios	Total	Indigenous	Agriculture	Total	Indigenous	Agriculture
**Social**						
One and a half times as many people in the catchment	-	-	-			
Twice as many people in the catchment				-	-	-
Twice the infrastructure	++	++	++	++	++	++
**Commercial**						
Twice as much clearing	-	-	+			
Four times as much clearing				-	—	+
Twice as much agriculture	-	—	+			
Four times as much agriculture				-	—	++
**Environmental**						
Water level dropped in the Daly (dry season)	—	—	—	—	—	—
Three quarters as many fish	-	-	-			
Half as many fish				—	—	—

## Discussion

Planning for multiple land uses requires navigating trade-offs between social, commercial, and environmental outcomes arising from different possible futures. Our study illustrates one method of incorporating stakeholder preferences, associated with development and conservation, into both the design and evaluation of land-use scenarios. Furthermore, our study presents an example of how scenarios can be designed using existing decision-support tools for optimal allocation of land uses, thereby ensuring multiple objectives are met while minimizing potential conflicts between competing interests [21].

The primary differences between our land-use scenarios were the extent and spatial placement of new land clearing for development and the associated environmental impacts, such as exceeding clearing levels of some vegetation types and potential reductions in dry-season flows of the Daly River due to increased water extraction. Scenario 1 had the fewest environmental impacts; for example, no clearing limits in the guidelines were exceeded. In contrast, Scenario 4 had the most impacts, involving both marginal and major exceedances to the clearing guideline limits and larger negative changes in satisfaction for Indigenous stakeholders related to amounts of clearing and agriculture. Agricultural precincts have the potential to concentrate intensive land uses. This concentration might result in benefits such as economies of scale of production and viable populations for attracting infrastructure investment (an outcome expected to increase satisfaction of both Indigenous and agricultural stakeholders), but also had associated negative impacts such as more clearing of specific vegetation types (Table 4).

A key goal for the Daly catchment is increased development [25, 26], which will necessarily result in further clearing; however, our analysis highlights that land-use changes associated with clearing for agriculture result in conflicts with residents’ preferences. Although the development scenarios resulted in increased development opportunities, and increases in satisfaction for stakeholders who might benefit, this came at the expense of environmental changes resulting in an overall decrease, on average, in stakeholder satisfaction, with the exception of increased infrastructure (Table 5). We found notable differences in the expected satisfaction with catchment changes between Indigenous respondents and those who earn an income from agriculture, particularly regarding economic factors, a finding that is in line with previous work in the region [9, 42–44]. Stoeckl et al. [9] demonstrated that, under a scenario of 5% annual agricultural industry growth, there were modest growths in income but incomes for Indigenous residents were expected to increase less than those of non-Indigenous residents. They also predicted noticeable hydrological and ecological impacts with the potential for reductions in well-being, particularly for Indigenous residents. Our results indicate that the potential increases in livelihoods associated with agricultural development might not balance the expected negative changes to satisfaction due to environmental and economic factors, and could lead to net reduction in satisfaction.

There are several limitations with our approach to estimating changes in satisfaction. First, the negative changes in satisfaction across most factors may relate to residents being satisfied with present aspects of life in the Daly (current reported satisfaction levels of 5, Fig 3); therefore any deviation from current aspects of life might result in negative changes to satisfaction. Second, our metrics of changes in Likert scale pose some difficulties in interpretation; a 1-point change may not indicate the same change in satisfaction across all parts of the Likert scale. For example, a 1-point change between 3 and 2 might be a threshold in which people become so dissatisfied they are willing to move, whereas a change between 5 and 6 might not be a significant change in residents’ overall wellbeing. Third, the survey did not present factors as trade-offs (e.g., how satisfied would you be with twice as much agriculture if it resulted in twice as much infrastructure?), but instead elicited satisfaction with changes in each factor independently [22]. Therefore, the analysis does not provide detail about what catchment residents would be willing to give up (e.g., changes in water level) to gain benefits associated with development (e.g., increased infrastructure).

These limitations could be directly addressed by presenting the land-use scenarios and satisfaction metrics to stakeholders to facilitate further exploration of why stakeholders are dissatisfied with suggested increases in clearing and agricultural development and what trade-offs they would be willing to make. For example, further engagement might confirm that stakeholders are satisfied with the current distribution of land uses in the catchment and that intensified agricultural development would negatively impact well-being. Alternatively, interactive exploration of scenarios results might reveal that stakeholders are willing to accept the negative changes in some factors to gain the benefits of other changes in factors. This type of stakeholder engagement could also be used to bring together stakeholder groups with potential opposing preferences, such as Indigenous and agriculture groups, to negotiate trade-offs and select scenarios that balance preferences. While there are a large number of residents within these stakeholder groups, representative bodies such as the Aboriginal Reference Group and NT Farmers Association could be engaged to negotiate for these larger groups of residents. Outcomes from further engagement could then inform the selection of a final land-use scenario that aligns the goals of further agricultural development with stakeholder preferences.

Our scenarios demonstrate that the suggested expansion of agricultural land uses in the Daly catchment could worsen residents’ life satisfaction and that future land-use decisions should consider potential trade-offs between increased income for some groups and decreased satisfaction with the environment. Negative changes in the environment will affect well-being factors associated with biodiversity, recreation, and cultural values which are more important to residents than commercial values [22]. Alternative development trajectories such as government-sector growth [9], which has lower associated environmental impacts, or carbon offsets [28] and payments for biodiversity conservation [45], which have associated environmental benefits, might be more socially acceptable for this region. Furthermore, given the direct link between the changes in agricultural land use and environmental factors such as water level and number of fish in the river, additional analyses to quantify the impacts of land-use scenarios on water extraction, using an existing evaluation tool [46], would contribute valuable insights.

## Conclusions

An important aspect of land-use planning is identifying alternative futures and building consensus among stakeholders around the best land-use options, taking into account perceptions of present and future well-being. This requires participation of stakeholders not only in the evaluation of potential scenarios but in the identification of explicit objectives that shape planning and scenarios. This collaborative approach can be challenging, particularly for regional-scale planning that involves diverse stakeholders and complex mixes of land uses.

One benefit of scenarios is that they can be used in participatory stakeholder workshops in which the different regional futures and expected changes in indicators of well-being can be explored and negotiated by different groups [12, 13]. Because our scenarios incorporated stakeholder preferences in the design and evaluation phases, the impacts of scenarios on stakeholders have been quantified and potential conflicts between stakeholders have been identified. This presents a unique set of information not commonly available to planners which can be used to facilitate further engagement to ideally create a consensus between groups but at the very least to acknowledge that differences exist.

## Supporting Information

S1 Fig Frequency (1–4) of agricultural land uses (perennial irrigation, annual irrigation and rainfed cropping) in subcatchments across the four scenarios. (DOCX)

S2 Fig Frequency (1–4) of agricultural land uses (perennial irrigation, annual irrigation and rainfed cropping) in subcatchments across the four scenarios for the two precincts (Douglas Daly precinct in the North and Katherine precinct in the South). (PDF)

S1 Table Numerical results for estimated changes in stakeholder satisfaction with changes in the Daly catchment. Reported satisfaction with changes in the catchment from [22] and estimated satisfaction from extrapolation (indicated with *) as they relate to A) 10% clearing scenarios and B) 20% clearing scenarios. Numbers based on averages from 0–10 Likert scales. Total, Indigenous, and Agriculture indicate results from all stakeholders and two separate groups of stakeholders (Indigenous, and those who earn an income from agriculture), respectively.
(PDF)

S2 Table Changes in satisfaction with potential future states of environmental characteristics compared to satisfaction with current states as they relate to 10% and 20% clearing scenarios. For characteristics for which respondents were prompted to report only a single satisfaction (e.g. water level dropped in the Daly (dry season) we used the most similar environmental characteristic with a linear extrapolation (e.g. number of fish) to estimate the satisfaction with the changed versus current status. A) 10% clearing scenarios and B) 20% clearing scenarios. Numbers based on average changes on 0–10 Likert scales. Total, Indigenous, and Agriculture indicate results from all stakeholders and two separate groups of stakeholders (Indigenous, and those who earn an income from agriculture), respectively.
(PDF)
