# Supplementary material for: Using Optimal Land-Use Scenarios to Assess Trade-Offs between Conservation, Development, and Social Values
Source: PLoS One. 2016 Jun 30;11(6):e0158350. doi: 10.1371/journal.pone.0158350 (PMC4928809; doi:10.1371/journal.pone.0158350)

**S1 Fig. Frequency (1-4) of agricultural land uses (perennial irrigation, annual irrigation and rainfed cropping) in subcatchments across the four scenarios.**
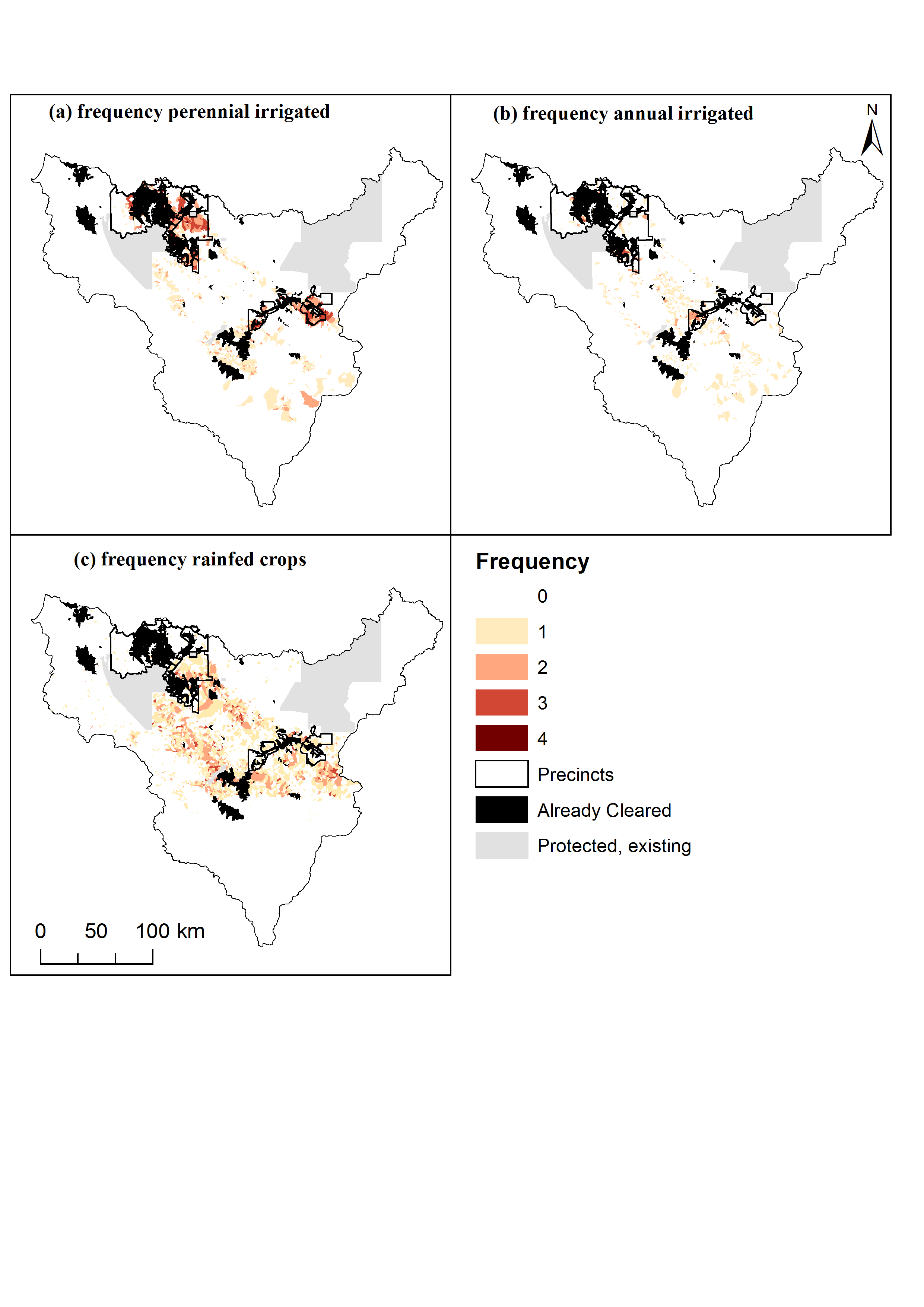

Supplement: S1 Fig — (DOCX) [file pone.0158350.s001.docx]
