# Supplementary material for: Using Optimal Land-Use Scenarios to Assess Trade-Offs between Conservation, Development, and Social Values
Source: PLoS One. 2016 Jun 30;11(6):e0158350. doi: 10.1371/journal.pone.0158350 (PMC4928809; doi:10.1371/journal.pone.0158350)

S2 Fig. Frequency (1-4) of agricultural land uses (perennial irrigation, annual irrigation and rainfed cropping) in subcatchments across the four scenarios for the two precincts (Douglas Daly precinct in the North and Katherine precinct in the South).

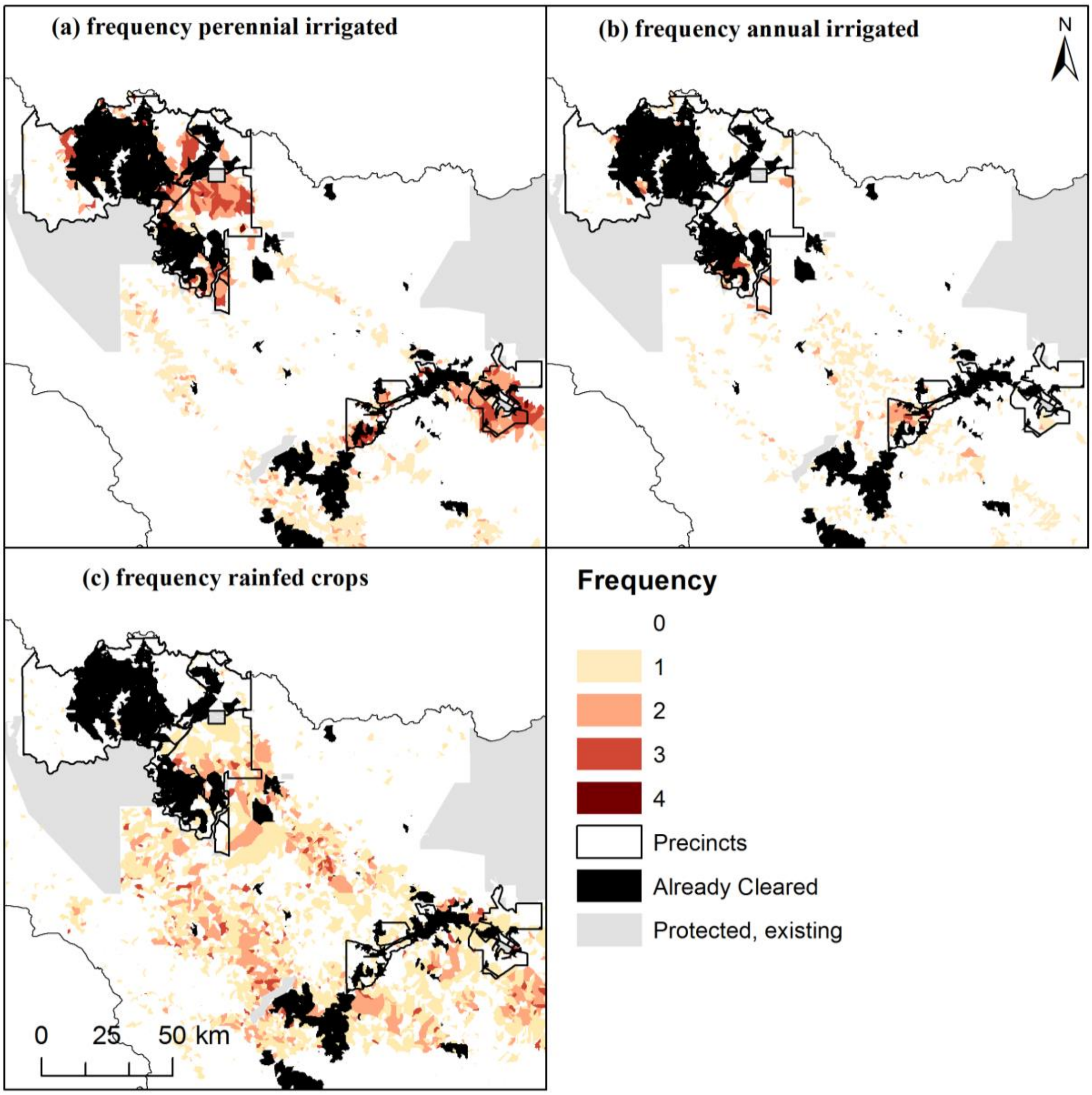

Supplement: S2 Fig — (PDF) [file pone.0158350.s002.pdf]
