# Supplementary material for: Using Optimal Land-Use Scenarios to Assess Trade-Offs between Conservation, Development, and Social Values
Source: PLoS One. 2016 Jun 30;11(6):e0158350. doi: 10.1371/journal.pone.0158350 (PMC4928809; doi:10.1371/journal.pone.0158350)
Supplement: S1 Table — Reported satisfaction with changes in the catchment from [22] and estimated satisfaction from extrapolation (indicated with *) as they relate to A) 10% clearing scenarios and B) 20% clearing scenarios. Numbers based on averages from 0–10 Likert scales. Total, Indigenous, and Agriculture indicate results from all stakeholders and two separate groups of stakeholders (Indigenous, and those who earn an income from agriculture), respectively. (PDF) [file pone.0158350.s003.pdf]

S1 Table. Numerical results for estimated changes in stakeholder satisfaction with changes in the Daly catchment. Reported satisfaction with changes in the catchment from [22] and estimated satisfaction from extrapolation (indicated with *) as they relate to A) 10% clearing scenarios and B) 20% clearing scenarios. Numbers based on averages from 0-10 Likert scales. Total, Indigenous, and Agriculture indicate results from all stakeholders and two separate groups of stakeholders (Indigenous, and those who earn an income from agriculture), respectively.

**A) 10% Clearing Scenarios**	**Total**	**Indigenous**	**Agriculture**
Water level dropped in the Daly (dry season)	2.47	2.02	3.12
Twice the infrastructure	5.93	6.05	6.15
Twice as much agriculture	3.96	2.59	6.04
Four times as much agriculture*	N/A	N/A	N/A
One and a half times as many people in the catchment *	4.13	4.13	4.43
Twice as many people in the catchment	N/A	N/A	N/A
Three quarters as many fish*	3.89	3.54	3.8
Half as many fish	N/A	N/A	N/A
Twice as much clearing	4.49	3.76	4.83
Four times as much clearing	N/A	N/A	N/A
**B) 20% Clearing Scenarios**	**Total**	**Indigenous**	**Agriculture**
Water level dropped in the Daly (dry season)	2.47	2.02	3.12
Twice the infrastructure	5.93	6.05	6.15
Twice as much agriculture	N/A	N/A	N/A
Four times as much agriculture*	3.7	2.1	7.75
One and a half times as many people in the catchment *	N/A	N/A	N/A
Twice as many people in the catchment	3.9	3.19	4.38
Three quarters as many fish*	N/A	N/A	N/A
Half as many fish	2.56	1.82	2.92
Twice as much clearing	N/A	N/A	N/A
Four times as much clearing	3.99	2.88	4.87

*estimated values
