# Supplementary material for: Using Optimal Land-Use Scenarios to Assess Trade-Offs between Conservation, Development, and Social Values
Source: PLoS One. 2016 Jun 30;11(6):e0158350. doi: 10.1371/journal.pone.0158350 (PMC4928809; doi:10.1371/journal.pone.0158350)
Supplement: S2 Table — For characteristics for which respondents were prompted to report only a single satisfaction (e.g. water level dropped in the Daly (dry season) we used the most similar environmental characteristic with a linear extrapolation (e.g. number of fish) to estimate the satisfaction with the changed versus current status. A) 10% clearing scenarios and B) 20% clearing scenarios. Numbers based on average changes on 0–10 Likert scales. Total, Indigenous, and Agriculture indicate results from all stakeholders and two separate groups of stakeholders (Indigenous, and those who earn an income from agriculture), respectively. (PDF) [file pone.0158350.s004.pdf]

S2 Table. Changes in satisfaction with potential future states of environmental characteristics compared to satisfaction with current states as they relate to 10% and 20% clearing scenarios. For characteristics for which respondents were prompted to report only a single satisfaction (e.g. water level dropped in the Daly (dry season) we used the most similar environmental characteristic with a linear extrapolation (e.g. number of fish) to estimate the satisfaction with the changed versus current status. A) 10% clearing scenarios and B) 20% clearing scenarios. Numbers based on average changes on 0-10 Likert scales. Total, Indigenous, and Agriculture indicate results from all stakeholders and two separate groups of stakeholders (Indigenous, and those who earn an income from agriculture), respectively.

**A) 10% Clearing Scenarios**	**Total**	**Indigenous**	**Agriculture**
Water level dropped in the Daly (dry season)*	-2.36	-2.74	-1.3
Twice the infrastructure*	1.47	1.59	1.64
Twice as much agriculture	-0.27	-1.09	1.7
Four times as much agriculture	N/A	N/A	N/A
One and a half times as many people in the catchment	-0.33	-0.33	-0.08
Twice as many people in the catchment	N/A	N/A	N/A
Three quarters as many fish	-0.94	-1.22	-0.62
Half as many fish	N/A	N/A	N/A
Twice as much clearing	-0.45	-0.25	0.04
Four times as much clearing	N/A	N/A	N/A
**B) 20% Clearing Scenarios**	**Total**	**Indigenous**	**Agriculture**
Water level dropped in the Daly (dry season)*	-2.36	-2.74	-1.3
Twice the infrastructure*	1.47	1.59	1.64
Twice as much agriculture	N/A	N/A	N/A
Four times as much agriculture	-0.53	-2.18	3.41
One and a half times as many people in the catchment	N/A	N/A	N/A
Twice as many people in the catchment	-0.56	-0.56	-0.13
Three quarters as many fish	N/A	N/A	N/A
Half as many fish	-2.27	-2.94	-1.5
Twice as much clearing	N/A	N/A	N/A
Four times as much clearing	-0.95	-1.13	0.08

*Estimated current satisfaction of status using most similar environmental factors, fish numbers and amount of agriculture.
